# Progressive Bidirectional Age-Related Changes in Default Mode Network Effective Connectivity across Six Decades

**DOI:** 10.3389/fnagi.2016.00137

**Published:** 2016-06-14

**Authors:** Karl Li, Angela R. Laird, Larry R. Price, D. Reese McKay, John Blangero, David C. Glahn, Peter T. Fox

**Affiliations:** ^1^Research Imaging Institute, University of Texas Health Science Center San AntonioSan Antonio, TX, USA; ^2^Department of Physics, Florida International UniversityMiami, FL, USA; ^3^Department of Mathematics and College of Education, Texas State UniversitySan Marcos, TX, USA; ^4^Department of Psychiatry, Yale University School of MedicineNew Haven, CT, USA; ^5^Olin Neuropsychiatry Research Center, Institute of Living, Hartford HospitalHartford, CT, USA; ^6^Genomics Computing Center, South Texas Diabetes and Obesity Institute, University of Texas Rio Grande Valley School of MedicineEdinburg, TX, USA; ^7^Research Service, South Texas Veterans Health Care SystemSan Antonio, TX, USA; ^8^Neuroimaging Laboratory, Shenzhen University School of MedicineShenzhen, Guangdong, China

**Keywords:** default mode network (DMN), structural equation modeling (SEM), meta-analytic connectivity modeling, functional connectivity (FC), normal aging

## Abstract

The default mode network (DMN) is a set of regions that is tonically engaged during the resting state and exhibits task-related deactivation that is readily reproducible across a wide range of paradigms and modalities. The DMN has been implicated in numerous disorders of cognition and, in particular, in disorders exhibiting age-related cognitive decline. Despite these observations, investigations of the DMN in normal aging are scant. Here, we used blood oxygen level dependent (BOLD) functional magnetic resonance imaging (fMRI) acquired during rest to investigate age-related changes in functional connectivity of the DMN in 120 healthy normal volunteers comprising six, 20-subject, decade cohorts (from 20–29 to 70–79). Structural equation modeling (SEM) was used to assess age-related changes in inter-regional connectivity within the DMN. SEM was applied both using a previously published, meta-analytically derived, node-and-edge model, and using exploratory modeling searching for connections that optimized model fit improvement. Although the two models were highly similar (only 3 of 13 paths differed), the sample demonstrated significantly better fit with the exploratory model. For this reason, the exploratory model was used to assess age-related changes across the decade cohorts. Progressive, highly significant changes in path weights were found in 8 (of 13) paths: four rising, and four falling (most changes were significant by the third or fourth decade). In all cases, rising paths and falling paths projected in pairs onto the same nodes, suggesting compensatory increases associated with age-related decreases. This study demonstrates that age-related changes in DMN physiology (inter-regional connectivity) are bidirectional, progressive, of early onset and part of normal aging.

## Introduction

### Motivation

The default mode network (DMN) is a widely studied, readily replicated neural network that has been implicated in a wide range of disorders affecting cognition, including neurological disorders (temporal lobe epilepsy, Ji et al., 2013; Parkinson's disease, Liu et al., 2013), psychiatric disorders (schizophrenia, Garrity et al., 2007; depression, Sheline et al., 2009), and developmental disorders (autism, Kennedy et al., 2006). Disorders exhibiting age-related cognitive decline, in particular, have been repeatedly and robustly demonstrated to show disordered processing (aberrant activity and connectivity patterns) within the DMN. The precuneus (Volkow et al., 2002) and posterior cingulate cortex (Minoshima et al., 1997; Johnson et al., 1998), two key hubs in the DMN, show significantly decreased cerebral glucose metabolism and blood flow and significantly increased variability in metabolic activity in patients with Alzheimer's disease. In turn, the coherence of their activity shows decreases in early Alzheimer's disease (He et al., 2007). Patients with amnestic mild cognitive impairment, a transitional stage between normal aging and Alzheimer's disease, also demonstrate aberrant connectivity when compared to controls (Bai et al., 2008), an abnormality that is correlated with cognitive task performance (Li et al., 2013). The DMN has been demonstrated to have unique metabolic characteristics, with a much higher rate of non-oxidative glucose consumption than other brain regions and networks (Vlassenko et al., 2010). This metabolic profile likely is due to the high tonic neural activity levels in the DMN and likely underlies its susceptibility to pathology (Sperling et al., 2009; Villain et al., 2010).

Age-related changes in DMN physiology have been reported in normal aging, as well as in pathology. Damoiseaux et al. (2008) compared healthy young adults (mean age = 22.8) with healthy older adults (mean age = 70.7), demonstrating significant decreases in DMN BOLD signal change in older subjects that were also correlated with decreased executive function (independent of age). Bernard et al. (2013) also reported overall decreases in the functional connectivity strength of large-scale resting state cortico-cerebellar networks in older adults (mean age = 64.6) compared to young adults (mean age = 22.8). However, it should be noted that both these studies compared cohorts at opposite ends of the age spectrum: young vs. old. To date, studies assessing DMN changes by sampling uniformly across the age spectrum, either in health or disease are lacking. The purpose of the investigations reported here was to determine age-related change patterns in the DMN in a large, cross-sectional sample of healthy normal subjects in a decade by decade manner.

### Background of the DMN

The concept of the DMN emerged from the early observation (Shulman et al., 1997; Binder et al., 1999; Mazoyer et al., 2001; Raichle et al., 2001) that while the spatial distribution of task-induced activation varied with the cognitive/sensory/motor demands of the specific tasks, task-induced decreases in cerebral blood flow were spatially consistent across tasks (task-negative regions). This was interpreted as indicating that a group of regions was tonically engaged during quiet rest, i.e., that the brain defaulted to using specific regions/network when not otherwise engaged. Various theories on the mental processes underlying DMN function have been proposed. From its task-negative nature it has been suggested that the DMN is responsible for non-goal-directed thought processes, monitoring of the environment and self, or perhaps monitoring of one's emotional state (Shulman et al., 1997). More recent works, however, have demonstrated the DMN's role in more goal-directed tasks. Meta-analysis of previous studies shows high involvement of the posterior cingulate cortex (PCC) in both episodic memory as well as processing emotionally salient stimuli (Laird et al., 2009). The DMN also demonstrates high overlap with regions underlying prospection, recall, and internal motivators (Spreng et al., 2009). The anterior network, including the medial prefrontal cortex, has been shown to play a role in linking visceral sensory stimuli with emotional behavior (Ongur and Price, 2000). Core areas of the DMN, including the posterior cingulate cortex, inferior parietal lobules, and medial temporal lobes, have also been shown to be involved in scene construction of past and fictitious events (Hassabis et al., 2007; Kim, 2012; Andrews-Hanna et al., 2014).

The DMN has been demonstrated via a variety of different modalities and analytical techniques. First analyzed using resting state fMRI connectivity in Greicius et al. (2003), DMN analysis using resting state fMRI data has since been extended to healthy adults (Damoiseaux et al., 2008; Bernard et al., 2013), children (Supekar et al., 2010), patients (Greicius et al., 2009), and even animals (Mantini et al., 2011). The DMN also consistently appears in independent components analysis of task-activation fMRI of both primary studies (Calhoun et al., 2009) and meta-analytic studies (Smith et al., 2009). In addition to functional changes with age and disease, structural changes in the DMN were demonstrated in patients with mild cognitive impairment (gray matter atrophy, Sorg et al., 2007). Physiological data demonstrates the overlap between the presence of amyloid plaques and the DMN (Buckner et al., 2005; Sperling et al., 2009), and plaque presence disrupts functional connectivity of the precuneus to other regions of the DMN (Sheline et al., 2010). Glycolytic index of brain metabolism shows a distribution similar to the DMN as well (Vlassenko et al., 2010). Taken together, the DMN can be considered a highly robust network observable by measurements from multiple modalities and of different natures.

### Meta-analytic model of the DMN

A comprehensive meta-analysis of the DMN was previously performed combining well-developed quantitative techniques with a vast database of functional activations to generate a candidate node-and-edge model of the DMN (Laird et al., 2009). Activation likelihood estimation (ALE; Turkeltaub et al., 2002) was performed on foci corresponding to task-induced deactivations to create a voxel-wise concordance map of the most probable regions of the DMN; nine functional regions (nodes) were identified. Building upon these identified regions, a meta-analytic connectivity model (MACM; Robinson et al., 2010) was constructed modeling inter-regional connectivity. Seeding each of the regions of interest (ROIs) to determine which pairs co-activated during task yielded a functional connectivity model with 13 connections. Two core hubs (exhibiting more extensive connectivity with other regions) in the posterior cingulate cortex and middle temporal gyrus were identified, suggesting their crucial role in the DMN. In this meta-analytic model, more extensively connected regions displayed lower levels of functional specialization while the more specialized regions exhibited a reduced degree of connectivity. The Laird MACM model provided a fully data driven, highly plausible candidate model for DMN connectivity, but did not quantify the strength of the connections between regions. Thus, additional analytic techniques are necessary to formally quantify DMN connectivity strength and its age-related changes.

### Modeling technique: Structural equation modeling

Structural equation modeling (SEM) is a general statistical analysis method that computes partial correlations among a set of mutually influential variables. Although not originally created for neural systems modeling, SEM has proven remarkably well-suited for this purpose (McIntosh and Gonzalez-Lima, 1994), and has a well-established literature base modeling connectivity in a variety of modalities and paradigms (Zhuang et al., 2005; Peltier et al., 2007; Laird et al., 2008; Price et al., 2009). SEM also offers the freedom to investigate networks in an exploratory manner wherein the model is iteratively evolved by adding or removing candidate interactions to best improve model fit. This allows identification of interactions potentially missed by an a priori model, and can also be used to improve a previously specified model (Price, 2013), a key feature required for ensuring that no strong connections are missed. SEM also models interactions in a simultaneous multivariate matrix, which allows modeling of mutual interactions between all nodes (as opposed to a pairwise approach). In addition, due to its rich history in psychometric analysis, SEM has a strong foundation that can be extended to model the interactions of a variety of physiological or neurological testing data upon the network, and is not restricted to solely modeling connectivity. SEM offers a statistical modeling framework to quantitatively track changes in DMN functional connectivity with age progression. Furthermore, it allows the evaluation of other factors (such as pathology and gender) and the strength of their interaction effects with the DMN.

Popular alternate approaches to modeling connectivity in neuroimaging datasets include casual modeling, most commonly dynamic causal modeling (DCM) and Granger causality. DCM is a modeling technique originally formulated to analyze functional connectivity modeled as fMRI responses to experimental inputs using an a priori specified biophysical model (Friston et al., 2003; Friston, 2009). More recently, DCM has been applied to modeling resting state fMRI data using a newer technique known as spectral DCM (Friston et al., 2014; Razi et al., 2015). However, spectral DCM for resting state data has only been performed with a specified model structure (Friston et al., 2014) or in an exploratory fashion with a more restricted set of four nodes (Di and Biswal, 2014; Sharaev et al., 2016). For the purposes of this study, we sought to examine a larger set of nodes identified by meta-analysis for comparison across age-groups, making SEM a more appropriate choice for the study. Granger causality is more similar to SEM in mathematical structure than DCM while retaining an emphasis on causal modeling. However, GC is more adapted to modeling neuronal causality, requiring high temporal resolution in the hundreds of milliseconds range (Kayser et al., 2009; Witt and Meyerand, 2009; Deshpande et al., 2010). The current study uses high resolution resting state data with a TR of 3 s, and would not be suited for analysis with Granger causality. A final commonly employed modeling technique is graph theory analysis, which can be applied to broadly test interactions between nodes within the DMN (Song et al., 2011), but lack a multivariate formalism that allows it to account for simultaneous effects from interactions between nodes.

### Goals of the present study

Here, we applied SEM to resting-state fMRI in a large healthy subject sample that spans a large age range (21–79 years). We report progressive alterations in the resting-state connectivity of the DMN using a previously published, meta-analytic model of the DMN (Laird et al., 2009) as a prior in six by-decade cohorts of healthy normal subjects. The primary physiological goal of this study was to characterize the progression of age-related adaptations in the DMN, a network strongly implicated in age-related cognitive decline. It was expected that a connectivity would should both decreases (decompensations) and increases (adaptations), but a specific pattern was not hypothesized. Prior studies have argued both for an anterior-to-posterior progression (Davis et al., 2008) and hemispheric asymmetries in aging (Cabeza, 2002; Dolcos et al., 2002). The methodology applied did not require a prediction of the anticipated pattern of change beyond that it would involve the DMN. The primary methodological goal of the study was to construct a connectivity model of the DMN using per decade functional resting state data and demonstrate its efficacy in investigating age-related changes in functional connectivity. As a previously established meta-analytic model served as a baseline to guide modeling, a secondary methodological goal was to assess the suitability of using meta-analytic modeling techniques to inform modeling of primary neuroimaging data.

## Methods

### Subjects

Data used in the present study are a subset of those acquired in the Genetics of Brain Structure (GOBS) project (1R01MH078111-01, David Glahn PI). GOBS participants are all of Mexican-American heritage living in the greater San Antonio region. All GOBS participants undergo extensive psychometric testing, including clinical diagnostic instruments as well as the imaging battery described below. From this data archive, 120 right-handed persons were selected at random spanning six decades (20–70s) of age, including 20 subjects (10 men and 10 women) per decade. All persons with known present or past neurological or psychiatric disorders or evidence of cognitive impairment by psychometric testing were excluded from the present analysis. Subjects in each decade cohort have similar levels of education. Intelligence quotient measures were also similar across decades using the Wechsler Abbreviated Scale of Intelligence II. Persons with systemic disorders common in this demographic (diabetes, hypertension, and hypercholesterolemia) were not excluded. See Table 1 for full breakdown of subject demographics. All participants provided written informed consent on forms approved by the institutional review board at the University of Texas Health Science Center at San Antonio (UTHSCSA).

**Table 1:** **Demographic information (gender, education in years, Wechsler Abbreviated Scale of Intelligence [WASI] II subtest IQ, global assessment of function score, and incidence of systemic disease) for subjects divided by decade**.

Decade	20 s	30 s	40 s	50 s	60 s	70 s
Gender (M:F)	10:10	10:10	10:10	10:10	10:10	10:10
Education (in years)	12.1	11.8	11.8	12.8	11.3	12.3
WASI II subtest IQ	87.2 ± 1.9	91.2 ± 2.3	83.5 ± 2.5	88.1 ± 3.3	85.6 ± 2.6	84.7 ± 2.7
Global assessment of function	81.0 ± 1.2	79.6 ± 2.4	84.5 ± 1.3	79.3 ± 2.6	79.9 ± 1.8	84.6 ± 1.0
Incidence of normal vs. Diabetes/Hypertension/Hypercholesterolemia	20–0/0/0	19–0/1/0	15–2/3/4	12–3/5/3	7–6/11/8	4–8/10/12

*Note that due to the WASI II subtest being administered in English while the participants were from Spanish speaking backgrounds, the test scores may skew lower. Subjects with a systemic disorder often were comorbid with other systemic disorders*.

### Image acquisition

Scanning was performed at the Research Imaging Center, UTHSCSA, on a 3T Siemens Trio scanner with an eight-channel head coil. High-resolution (isotropic 800 μm) 3D TurboFlash T1-weighted anatomic images were acquired for each subject using a retrospective motion-corrected protocol (Kochunov et al., 2006) with the following parameters: echo time (TE)/ repetition time (TR)/time for inversion (TI) = 3.04/2,100/785 ms and flip angle = 13°. Whole brain, resting state functional imaging was performed using a gradient-echo echoplanar imaging (EPI) sequence sensitive to the BOLD effect (TE/TR = 30/3000 ms; flip angle = 90°; isotropic 1.72 mm^2^). The 7.5-min resting state protocol included 43 slices acquired parallel to the anterior commissure and posterior commissure plane. During the resting state scan, subjects were instructed to lie in dimmed light with their eyes open and try not to fall asleep.

### Regions of interest selection

The DMN model constructed by Laird et al. (2009) using activation likelihood estimation (ALE) and meta-analytic connectivity modeling (MACM) was adopted as our starting model. The Laird MACM model consisted of nine nodes connected by 13 edges. Data from the BrainMap® database (Fox and Lancaster, 2002) were used to construct the model, including 119 published deactivation contrasts from 62 papers, representing 840 individual subjects and 1056 coordinate brain locations. The nodes included: precuneus (pC), left middle frontal gyrus (LMFG), left inferior parietal lobule (LIPL), right inferior parietal lobule (RIPL), posterior cingulate cortex (PCC), middle prefrontal gyrus (MPFG), left middle temporal gyrus (LMTG), right middle temporal gyrus (RMTG), and ventral anterior cingulate cortex (vACC). Table 2 details the coordinate locations of the centers of mass of DMN nodes in Talairach space (Talairach and Tournoux, 1988).

**Table 2:** **Regions of interest with coordinates**.

**Region of interest**	**(x, y, z)**
Precuneus (pC)	(−4, −58, 44)
Posterior Cingulate Cortex (PCC)	(−4, −52, 22)
Ventral Anterior Cingulate (vACC)	(2, 32, −8)
R Inferior Parietal Lobule (RIPL)	(52, −28, 24)
Medial Prefrontal Gyrus (MPFG)	(−2, 50, 18)
R Middle Temporal Gyrus (RMTG)	(46, −66, 16)
L Middle Frontal Gyrus (LMFG)	(−26, 16, 14)
L Inferior Parietal Lobule (LIPL)	(−56, −36, 28)
L Middle Temporal Gyrus (LMTG)	(−42, −66, 18)

*Regions were derived from a meta-analysis of deactivations across the BrainMap database (Laird et al., 2009)*.

### Data pre-processing

Image analysis was performed with FMRIB's Software Library (FSL) tools (www.fmrib.ox.ac.uk/fsl). Preprocessing for resting state data included motion correction, brain extraction, spatial smoothing (5 mm FWHM Gaussian kernel), and two runs were performed with high-pass temporal filtering (100 s) (http://fsl.fmrib.ox.ac.uk/fsl/fslwiki/FEAT/UserGuide). FSL uses a local fit of a straight line smoothed by Gaussian weighting to remove low frequency artifacts as opposed to a sharp rolloff frequency filter to avoid introducing additional autocorrelation into the data (http://fsl.fmrib.ox.ac.uk/fsl/fslwiki/FEAT/UserGuide).

Head motion has previously been demonstrated to be a small but significant source of variance in functional connectivity. In particular, the DMN has been shown to exhibit decreased within-network functional connectivity with increasing motion (Van-Dijk et al., 2012). This can be corrected by the inclusion of a number of motion parameters that can include simple x, y, z translations as well as additional temporal derivatives of the parameters to further correct for delays in effects (Satterthwaite et al., 2013). Motion correction was applied to each time series extracted per ROI x subject via FSL's MCFLIRT tool, which takes the middle volume of the time series and compares adjacent time points successively to estimate rotation and translational matrices to correct for the effects of motion using multiple sequential time points as reference (Jenkinson et al., 2002). Each subject's fMRI volumes were linearly aligned using FSL's FRMIB Linear Image Registration Tool (FLIRT; Jenkinson and Smith, 2001), first aligning each subject's resting state scan to their high-resolution neuroanatomic scan, and then to a common Talairach space. In all, this created six sets of 20 4D data sets (6 age groups × 20 subjects per group × 150 scans per subjects = 18,000 images).

Many studies have also demonstrated the effects of including average white matter and cerebral spinal fluid signals as additional regressors that can account for the effects of motion as well as physiological effects such as respiration and cardiac pulsation (Windischberger et al., 2002; Lund et al., 2006). Data cleaning processes that include motion correction generally yield better temporal signal-to-noise ratio and more consistent connectivity maps. However, most studies evaluate the effectiveness of the methods through voxel-based approaches such as independent components analysis (Murphy et al., 2011, 2013; Bright and Murphy, 2015). This results in reduction in the noisiness of connectivity between voxels, generally achieved by eliminating motion induced falsely coactivating voxels on the periphery of each cluster. By contrast, an ROI based approach pre-averages a cluster of voxels and hence pre-smooths the defined area. Fluctuations in signal at the voxel level are unlikely to impact the overall time course signal. The impact of using voxel-based signal denoising methods on evaluating connectivity strength between select gray regions using SEM is much less understood, especially since regression of white matter and CSF generally results in decreased gray matter connectivity mostly with deep tissue and ventricles (Griffanti et al., 2015). A common concern cited when using regressors to remove the effects of noise is the potential for removal of true neural signals along with the noise (Power et al., 2015), and in the case of this study, the efficacy of noise-correction methods applied to region based data analyzed with SEM is not well-understood. As such, additional preprocessing of the data was also performed using white matter and cerebral spinal fluid signals (each isolated using FSL's FMRIB's Automated Segmentation Tool [FAST]) as confounds that were regressed from the resting state scans to compare with the non-regressed data. SEM derived path coefficients using this data showed decreases of varying magnitude in all paths in the DMN as well as motor network when compared to unscrubbed data. However, almost all age-related trends continue to hold (see Supplementary Table I), and due to the unclear efficacy of applying these regressors to ROI based analysis with SEM (instead of voxel based ICA), the data presented above and discussed below are without these confounds regressed.

Time series data were then extracted from the nine DMN ROIs using FSL's Featquery tool with 12 mm radius spherical ROI masks created in MANGO (Multi-Image Analysis GUI) developed at the Research Imaging Institute at UTHSCSA (http://rii.uthscsa.edu/mango/). Following time series extraction, additional filters were applied removing time points showing high changes compared to the time series average to exclude motion-induced time points that could not be fully corrected by MCFLIRT. Specifically, time points that displayed significant sudden (from time point to time point) signal deviation across multiple ROIs (more than 7 standard deviations across at least 2 ROIs, 6 SDs across 3 ROIs, 5 SDs across 4 ROIs, 4 SDs across 5 ROIs, 3 SDs across 6 ROIs) were removed.

### Structural equation modeling

#### Unified SEM approach

**Figure 1:**
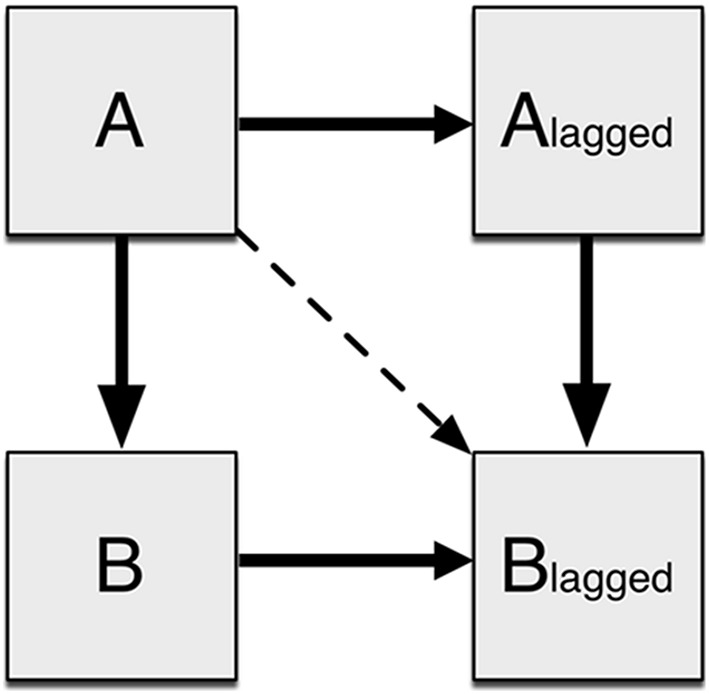
**Connectivity between two regions as modeled by SEM**. An ROI is modeled as two observed variables: the original time series and the time series offset by one time point. A loads onto B with both the original and offset time series. Additionally A loads on to the offset B time series to account for any delayed effects.

Following extraction, the data were normalized for each Subject × ROI to a mean of zero and a variance of one to reduce bias. DMN connectivity was assessed using a unified SEM approach (Kim et al., 2007), implemented in Amos 22.0 (IBM, Inc.). The distinction between traditional SEM and unified SEM is the inclusion of additional variables that improve the temporal representation of fMRI data via multivariate autoregressive modeling. Due to the strong autocorrelations present in fMRI time series data (Friston et al., 1995), each ROI was represented in the SEM by two variables: one of the time series extracted from the data set, and the other a lag-1 version that has each time point offset by one (Kim et al., 2007). Each lagged variable represents the next time point, and A → A_lagged_ (Figure 1) captures the autoregressive component of the time series variance. Partial autocorrelations in time series data corrected with the high-pass temporal filter were estimated using sample covariances using the software R. Successively higher order autoregressive models were fitted to a maximum of a lag-5 model. Time series data from fMRI studies generally do not have higher order than lag-1, but up to 5 were tested to ensure accuracy. As the study used resting state scans with no inherent block structure, it was expected that there would not be later lag factors with significant impact. These analyses demonstrated, in agreement with prior results (Bullmore et al., 1996), that lag-1 autocorrelations explained over 36% of the variance in FMRI data on average, while lag-2 and higher explained 5% or less with diminishing returns for each additional lag factor. Hence, unified SEM included an added set of variables based on a multivariate autoregressive lag-1 model. To assess the interactions between two brain regions A and B, four variables were created with three possible paths representing both contemporaneous (unlagged) effects as well as any longitudinal (lagged) effects. For example, ROI A loading on ROI B was modeled as A_unlagged_ loading on B_unlagged_, A_lagged_ loading on B_lagged_, and A_unlagged_ loading on B_lagged_ (Figure 1). The most informative effect is A_lagged_ loading on B_lagged_, as it captures the effects of A on B after having considered delayed effects as well as the effects of autocorrelation. This general procedure for examining the relationships between two variables was then extended to simultaneously model all interactions for all variables.

#### Laird MACM model fitting

The Laird MACM model was tested using SEM combining the unified SEM approach described above with the published edges between nodes. Five of the paths in the MACM model were specified to be bidirectional, given that both regions in the path appeared in one-another's MACM maps. Thus, these paths were tested in a non-recursive SEM as a feedback loop as well as recursively using a unidirectional path to determine which modeling approach yielded the best fit for the resting state data. In all cases, the recursive SEM with unidirectional paths produced the model with lowest RMSEA and highest CFA. Finally, model fit for the entire subject pool and per age group was assessed to determine the robustness of the Laird MACM model fit to resting state fMRI data.

#### Exploratory modeling

To construct a model that provides optimal fit, exploratory SEM using published nodes and every possible edge was also performed. Our exploratory model fitting protocol followed guidelines established from research in the information-theoretic and Bayesian modeling fields (Leamer, 1978; Raftery, 1993; Burnham and Anderson, 2002). Specifically, we employed the Kullback-Leibler distance measure (Kullback and Leibler, 1951) as incorporated into the information theoretic measure the Browne-Cudeck Criterion (BCC; Browne and Cudeck, 1993), to identify the model with the highest probability of being the correct model. The BCC was developed specifically for covariance structure modeling and imposes a greater penalty for model complexity than does the Akaike Information Criterion (AIC) and the Bayesian Information Criterion (BIC). The BCC is defined as:

$$BCC=\hat{C}+2q\frac{\Sigma_{g\;=\;1}^{G}b^{\left(g\right)}\frac{p^{\left(g\right)}(p^{\left(g\right)}\;+\;3)}{N^{\left(g\right)}-p^{\left(g\right)}-2}}{\Sigma_{g\;=\;1}^{G}b^{\left(g\right)}(p^{\left(g\right)}\;+\;3)}$$

Where, Ĉ is the minimum value of the discrepancy function, *q* is the number of parameters in the model, p is the number of sample moments in all groups combined, *b*^(*g*)^ equals the total sample size (*N*) times the ratio of the sample size in a group *N*^(*g*)^ to the total sample size (N), *p*^(*g*)^ is the number of variables in an observed group *N*^(*g*)^, and *G* is the number of groups in the model. Of particular relevance, the exploratory strategy we employed provides a mechanism for the prevention of overfitting (a challenge in specification search procedures with high-dimensional data structures, Hastie et al., 2009; Gelman, 2014). Exploratory modeling began with a null model with only the unlagged version of each ROI loading on the lagged version, the model representation that each region has no significant correlation with the activity of any other region. Fit statistics for the baseline model is provided in Table 3. Model improvement evolved through evaluating improvement in the root mean square error of approximation (RMSEA; Steiger, 1990) and Browne-Cudeck Criterion (BCC) with successive additions. Modification indices (an index that is a lower bound estimate of the improvement in chi-squared statistic when a parameter is allowed to be unconstrained instead of zero) were used to identify candidate connections, and the top five choices were tested for the three-path A to B and B to A connection that yielded the greatest improvement to the model. RMSEA is defined as:

$$\text{RSMEA}=\surd (\frac{\text{max}(\chi^{2}-df,0)}{n*df})$$

where χ^2^ is the chi-squared statistic for the goodness of fit of the model, *df* is the degrees of freedom in the model, and *n* is the number of samples. RMSEA has 90% confidence interval upper and lower limits found by first solving the non-central chi-squared distribution:

$$\begin{array}{l} \Phi \left(\chi^{2}|\delta_{U},d\right)=0.05\\ \Phi \left(\chi^{2}|\delta_{L},d\right)=0.95\\ \text{ HI90}=\surd (\frac{\delta_{U}}{n*d})\\ \text{ LO90}=\surd (\frac{\delta_{L}}{n*d}) \end{array}$$

The RMSEA was selected to be the primary fit criterion because it is not as sensitive to the effects of sample size. An RMSEA of 0.05 or 0.08 has typically been deemed as indicative of a reasonably good fit to the data (Browne and Cudeck, 1993). In order to achieve a satisfactory level of fit without creating an overly complex model, an RMSEA 90th percentile confidence interval upper bound of 0.08 for at least half the age groups was selected to be the criterion for a final model. This criterion was selected to ensure that the resulting model fits the cohorts reasonably well without being overly skewed by one or two age groups that require overly complex fits.

**Table 3:** **Iterative process to building the exploratory model**.

**Addition**	**χ^2^**	***DF***	**RMSEA**	**BCC**	**Modification index**
None	108963.189	864	0.0841	113770.189	n/a
pC –> PCC	90425.675	846	0.0773	90788.013	1621.349
PCC –> pC	90744.040	846	0.0775	91106.378	1621.349
pC –> LMTG	96708.717	846	0.0800	97071.055	1304.231
LMTG –> pC	96623.581	846	0.0799	96985.919	1304.231
pC –> RMTG	97937.403	846	0.0805	98299.740	1196.528
RMTG –> pC	97785.355	846	0.0804	98147.693	1196.528
**pC –> PCC ADDED**					
pC –> LMTG	78171.204	828	0.0726	78569.775	1304.231
LMTG –> pC	78286.068	828	0.0727	78684.639	–
pC –> RMTG	79399.889	828	0.0732	79798.460	1196.528
RMTG –> pC	79247.842	828	0.0731	79646.413	–
RIPL –> LIPL	83086.561	828	0.0746	83485.133	1174.327
LIPL –> RIPL	83069.857	828	0.0745	83468.428	1174.327

*The top five possible connections between two ROIs were identified using modification indices (only three are shown per step here) and the three path connection as shown in Figure 1 was tested for both A->B and B->A. In the first step, pC - -> PCC was determined to improve RMSEA the most, and was added to the model, and a second iteration began with the connection included. LMTG - -> pC and RMTG - -> pC did not show up in the top modification indices (indicated by the dash), but their model improvements were also tested for thoroughness*.

#### DMN age effects

Path coefficients vary around an average value (i.e., each path coefficient produced by SEM is associated with a regression error). The standard error of the path coefficient for a single subject is relatively large compared to the average. Using the model generated via exploratory SEM, aging trends were assessed by correlating the path coefficients observed within each age group. For the purpose of a negative control, a second set of meta-analytically derived ROIs supporting motor execution (Laird et al., 2008) were analyzed, including the primary motor cortex, ventral premotor, secondary somatosensory cortex, posterior parietal cortex, and cerebellum. The motor network was chosen because of a rich literature of investigations demonstrating age-related changes in motor network behavior (Wu and Hallett, 2005; Naccarato et al., 2006; Graziadio et al., 2015) and the analysis was intended as a comparison to the DMN network to determine if similar age-related patterns would be observed.

## Results

### MACM model fit

The Laird MACM model was specified *a priori*, and was tested for model fit using the unified SEM approach. Overall, the Laird MACM model did not fit any of the individual age groups with acceptable levels of fit based on RMSEA, though it provided a reasonable fit of the entire subject pool as a whole (Tables 4, 5). Because of its non-significant fit to each individual age group (no decade cohort had an RMSEA < 0.122), the Laird MACM model was not considered a sufficiently well-fitting candidate model to test age-related changes in functional connectivity strength, as relatively large amounts of covariances were not explained by the model across every age group.

**Table 4:** **Fit statistics of the baseline (no connectivity between regions), Laird MACM, and exploratory SEM models for the entire subject pool**.

**Fit statistic**	**Baseline**	**Laird MACM**	**Exploratory SEM**
RMSEA	0.084–0.084	0.051–0.052	0.030–0.031
Browne-Cudeck criterion	109289.292	31107.005	11961.529
Comparative fit index	0.169	0.770	0.918
χ^2^	108963.189	30309.863	11164.387
Degrees of freedom	864	630	630
Sample size	17832	17832	17832

*Across every fit statistic assessed, the exploratory SEM model provides fits that approach acceptable levels. The Laird MACM model, while not fitting as well as the exploratory SEM model, does fit reasonably well according to RMSEA*.

**Table 5:** **RMSEA 90% confidence intervals for the MACM model and exploratory model across age groups**.

**Decade**	**Baseline**	**Laird MACM**	**Exploratory SEM**
RMSEA (20s)	0.230–0.235	0.143–0.149	0.085–0.091
RMSEA (30s)	0.215–0.220	0.126–0.132	0.072–0.078
RMSEA (40s)	0.191–0.196	0.122–0.128	0.083–0.089
RMSEA (50s)	0.207–0.212	0.123–0.129	0.080–0.086
RMSEA (60s)	0.212–0.217	0.138–0.144	0.071–0.077
RMSEA (70s)	0.210–0.215	0.127–0.133	0.070–0.076

The MACM model is a distinct improvement over the baseline model, but lacks a few key paths that would greatly improve model fit, as indicated by the superior fit of the exploratory SEM. Each decade cohort was fit to just under 3000 sample points (150 time points ^*^ 20 subjects—discarded time points due to motion).

### Exploratory model fit

**Figure 2:**
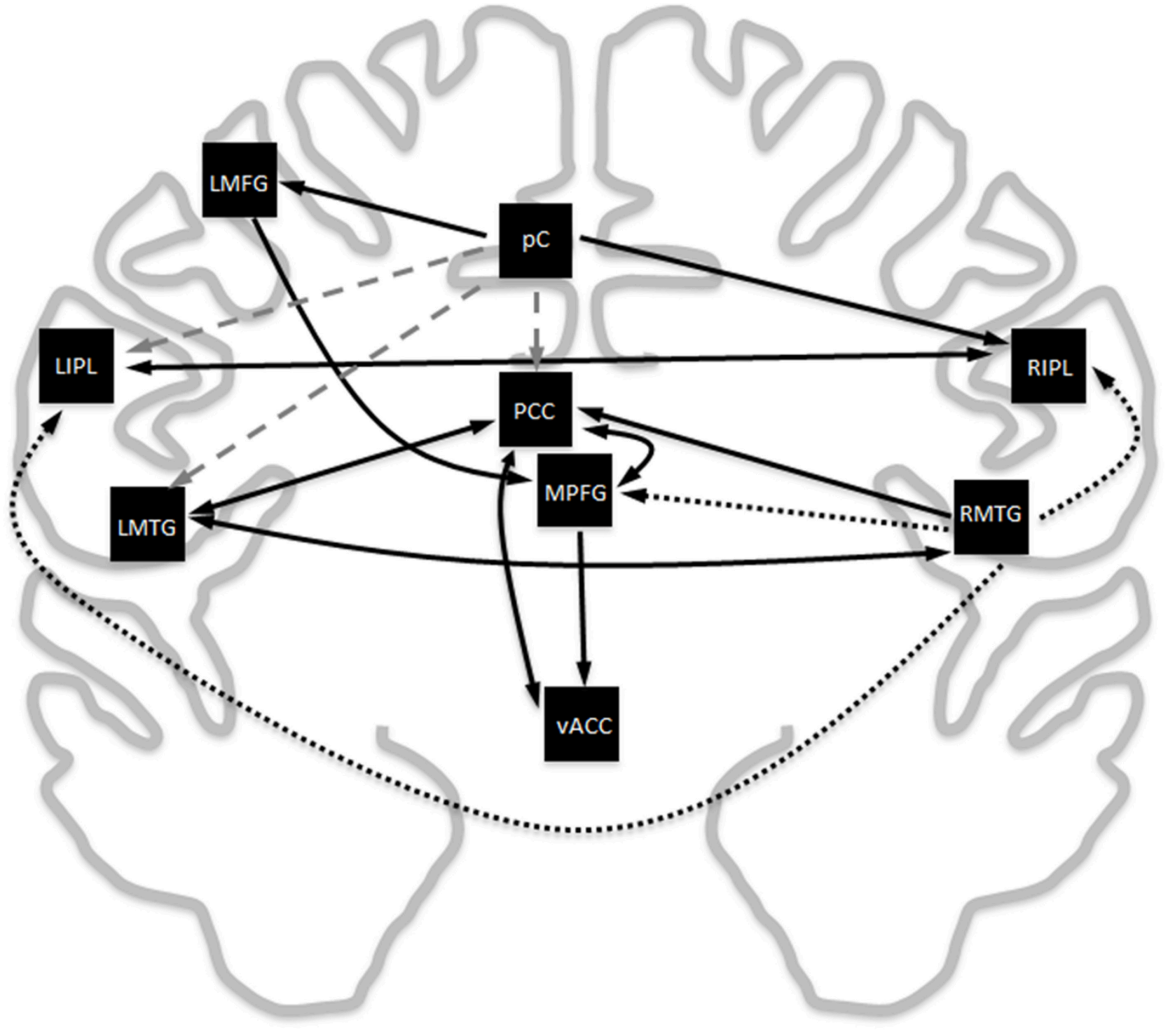
**Comparison of the Laird MACM model with the data-derived exploratory SEM model**. Black solid lines are paths that overlap between the two models, black dotted lines are paths that are exclusively in the MACM model, and gray dashed lines are paths that exclusively appear in the exploratory SEM model. Ignoring directionality, there is an overlap in 10 of the 13 paths. The probability for this to have occurred by random chance is 0.023%.

Exploratory modeling iteratively improving upon model fit resulted in 13 connections for a total of 39 paths. The final model fit the data as a group well (χ^2^ = 11164.4, *df* = 630, RMSEA = 0.030, BCC = 11961.5), and fit the decade cohorts reasonably well (highest RMSEA upper bound was 0.091). The resulting model was very similar to the meta-analytic connectivity model (MACM) previously established (Figure 2). Given that there are 36 possible connections between regions ignoring directionality, the probability of 10 of the 13 paths in the MACM model showing up in the exploratory model by random chance is 0.023%, suggesting that MACM provided a reasonable *a priori* model that needed only minor modifications to fit the primary data well. The key difference between the two models is the relative importance of the right middle temporal gyrus vs. the precuneus.

### Age-related trends

**Figure 3:**
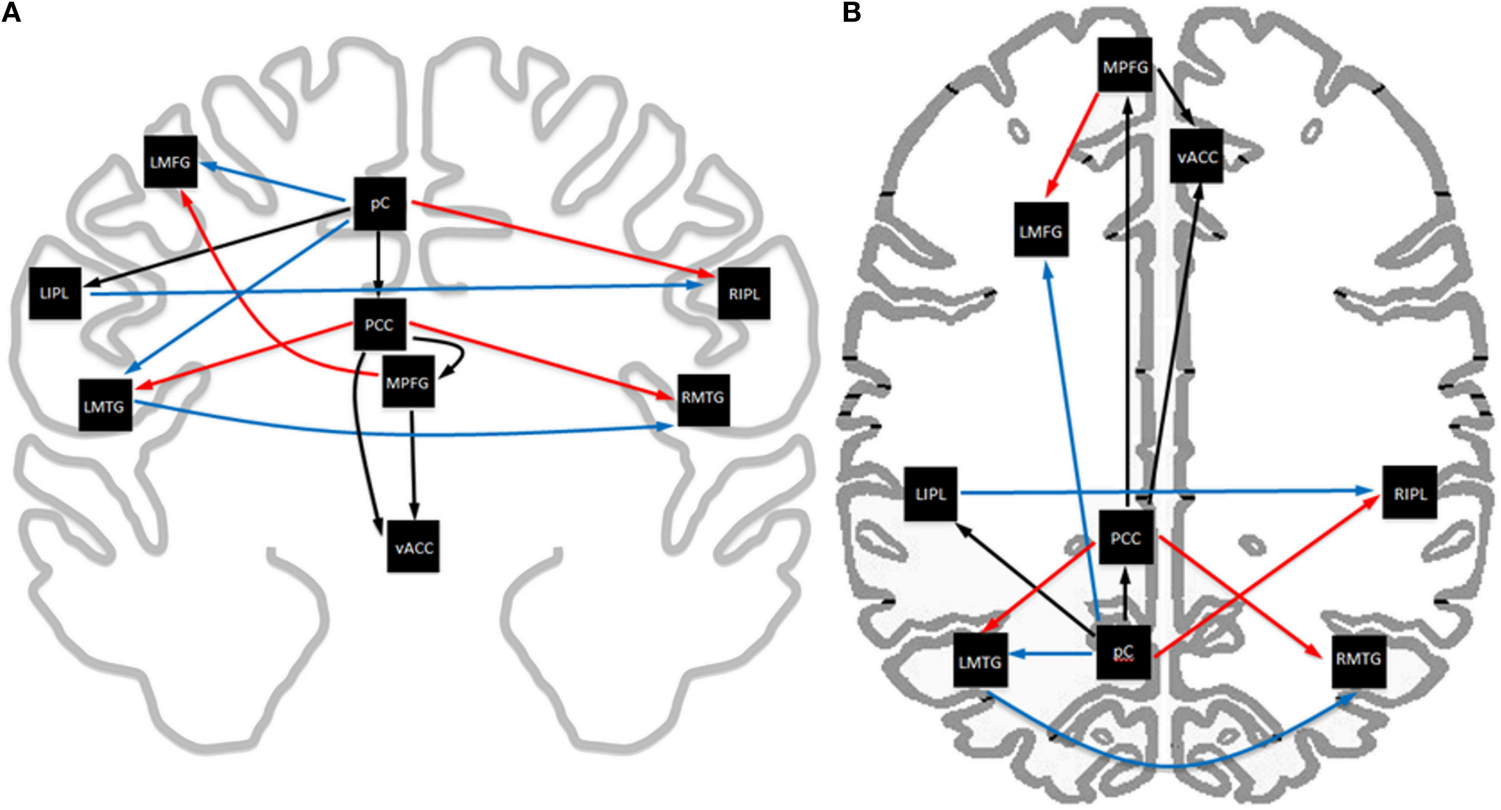
**Axial (A) and coronal (B) view of the exploratory SEM model of resting state data**. Age-related trends in functional connectivity strength are highlighted in color, with red indicating increasing strength with age, blue indicating decreasing strength with age, and black indicating no significant change with age.Midline paths show little change with age while lateralized regions show significant changes with age. Connectivity in the posterior circuit show extensive changes with age.

**Figure 4:**
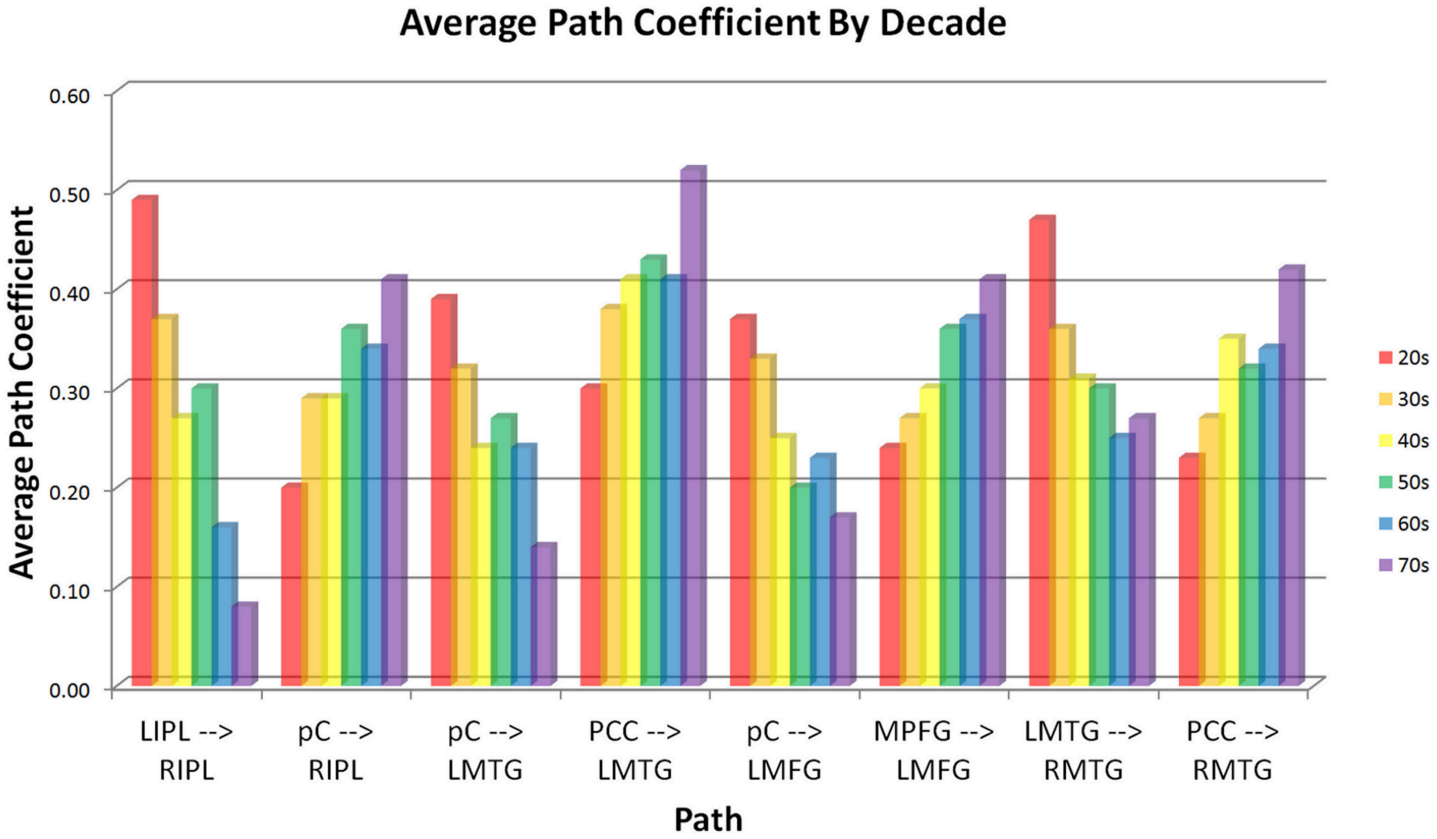
**Connectivity of paths showing age-related changes by decade**. Paths are paired by receiving region. Each node receives one path that increases in strength with age and one that decreases with age. Effects are strongly linear (lowest correlation between average age of each decade cohort vs. average path coefficient of the cohort is *r* = −0.89, significant at *p* = 0.05 level).

Analysis of the path coefficients revealed that eight of the thirteen connections found in the DMN showed significant linear age-related changes (Figure 3 and Table 6). The four regions receiving connections that exhibited age-related changes received them in pairs that increased and decreased in strength with age (Figure 4). The regions receiving age-varying paths represent four out of the five lateralized regions in this study (LMFG, RIPL, LMTG, RMTG; LIPL is the exception). Paths that project to midline regions did not show significant path coefficient changes with age. The total increases or declines from the twenties to the seventies varied by path, but were 10–20 times the standard error of the regression weight estimates (0.013 to 0.021 range). Due to the far lower standard error of the regression weights of the decade cohorts compared to per subject regression weights, it is a more reliable indicator, and age-related correlations were performed using decade path coefficients instead (per subject correlations do show similar effects). Age-related trends were similar for both males and females across all paths except between MPFG and LMFG, where females demonstrated significant monotonic increases (0.11, 0.24, 0.30, 0.34, 0.36, 0.47 by decade, *r* = 0.97), while males largely stayed level (0.37, 0.34, 0.35, 0.36, 0.38, 0.35 by decade; *r* = 0.10).

**Table 6:** **Path coefficients (per decade cohort) for the exploratory SEM model arranged by hub**.

**Path**	**Path Coefficient (By Decade)**	**Pearson r Correlation with Decade**
**pC → LMFG**	**0.37 | 0.33 | 0.25 | 0.20 | 0.23 | 0.17**	−**0.93**
**pC → RIPL**	**0.20 | 0.29 | 0.29 | 0.36 | 0.34 | 0.41**	**0.94**
pC **→** LIPL	0.42 | 0.39 | 0.38 | 0.37 | 0.47 | 0.44	0.46
**pC → LMTG**	**0.39 | 0.32 | 0.24 | 0.27 | 0.24 | 0.14**	−**0.93**
pC **→**PCC	0.64 | 0.61 | 0.61 | 0.59 | 0.62 | 0.60	−0.59
**PCC → LMTG**	**0.30 | 0.38 | 0.41 | 0.43 | 0.41 | 0.52**	**0.91**
**PCC → RMTG**	**0.23 | 0.27 | 0.35 | 0.32 | 0.34 | 0.42**	**0.92**
PCC **→**MPFG	0.47 | 0.52 | 0.47 | 0.48 | 0.41 | 0.45	−0.61
PCC **→**vACC	0.23 | 0.25 | 0.24 | 0.27 | 0.19 | 0.18	−0.41
MPFG **→**vACC	0.42 | 0.39 | 0.31 | 0.31 | 0.44 | 0.42	0.15
**MPFG → LMFG**	**0.24 | 0.27 | 0.30 | 0.36 | 0.37 | 0.41**	**0.99**
**LIPL → RIPL**	**0.49 | 0.37 | 0.27 | 0.30 | 0.16 | 0.08**	−**0.97**
**LMTG → RMTG**	**0.47 | 0.36 | 0.31 | 0.30 | 0.25 | 0.27**	−**0.89**

*The precuneus is the core hub with five connections exiting followed by the posterior cingulate cortex with four. The medial prefrontal gyrus has two connections with other nodes in the anterior network. The two remaining paths are connections between bilateral regions. Bolded rows are paths that show significant correlation with age. Unbolded paths demonstrate neither significant linear trends with age nor vary significantly across the age-span. Standard errors range from 0.013 to 0.021*.

### Comparison to motor ROIs

Age-related changes in connectivity between motor ROIs were minimal (Table 7); in homologous pairs, a decreasing trend was only observed between the secondary somatosensory cortices. The other pairs showed little change in connectivity strength with age. Notably, even extending the analysis to all possible pairings of the regions (45 total), no path was observed to increase in strength with age.

**Table 7:** **Correlation coefficients of the 13 strongest pairs within the motor control dataset**.

**Path**	**Correlation Coefficient (By Decade)**
LCer < - -> RCer	0.58 | 0.61 | 0.58 | 0.68 | 0.60 | 0.60
LM1 < - -> RM1	0.54 | 0.56 | 0.45 | 0.53 | 0.54 | 0.44
LPMv < - -> RPMv	0.54 | 0.39 | 0.31 | 0.45 | 0.42 | 0.32
LS2 < - -> RS2	0.60 | 0.39 | 0.33 | 0.46 | 0.38 | 0.13
LPPC < - -> RPPC	0.65 | 0.58 | 0.62 | 0.68 | 0.65 | 0.64
RM1 < - -> RPPC	0.48 | 0.50 | 0.48 | 0.57 | 0.60 | 0.45
LM1 < - -> LPPC	0.54 | 0.55 | 0.45 | 0.48 | 0.56 | 0.56
RM1 < - -> LPPC	0.45 | 0.50 | 0.40 | 0.48 | 0.52 | 0.49
LM1 < - -> RPPC	0.43 | 0.47 | 0.42 | 0.48 | 0.53 | 0.41
RPMv < - -> RS2	0.59 | 0.42 | 0.39 | 0.50 | 0.41 | 0.31
LPMv < - -> LS2	0.48 | 0.39 | 0.36 | 0.46 | 0.49 | 0.37
RM1 < - -> RS2	0.59 | 0.42 | 0.39 | 0.50 | 0.41 | 0.31
LM1 < - -> RS2	0.47 | 0.42 | 0.29 | 0.43 | 0.39 | 0.23

*While one pair (LS2 and RS2) shows significant decreases with age and other pairs show nearly significant decreases with age as well, no pair (including ones not shown) show increases in strength with age*.

### Subject motion correction

Subject motion did not differ significantly across decades, although it was highest in subjects in their 70 s (see Supplementary Table II). This trend was not significant using a Pearson r test for either the absolute displacement (*r* = 0.09, *DF* = 118, *p* = 0.33) or relative displacement (*r* = 0.14, *DF* = 118 *p* = 0.13) ANOVA tests of displacement differences also indicate no significant differences across decades (*F* = 0.97, *p* = 0.44 for absolute displacement; *F* = 0.81, *p* = 0.55 for relative displacement; *F*_critical_ = 2.29).

## Discussion

A primary methodological goal of this study was to demonstrate the efficacy of using SEM to elucidate age-related changes in neural systems. This was strongly confirmed, as a well-fitting model was constructed with robust linear changes in connectivity strengths with age observed in multiple paths, also confirming the primary physiological goal. A secondary methodological goal was to determine the suitability of meta-analytic modeling to guide and inform SEM (and other node-and-edge modeling constructs). While a shift in the center of activity was observed, the primary data driven exploratory model showed overall strong agreement with the Laird MACM model, suggesting that meta-analytically generated models may provide a useful source for baseline models that are not biased by a limited sample of primary data.

### Age-related effects

Path coefficients significantly correlated with age were observed in 8 of the 13 paths in the SEM exploratory model (Figure 3). Changes with age were restricted to the paths involving lateralized regions while all four medial paths demonstrated no correlation with age. One of the most notable effects was the paired increases and decreases of connections with age (Figure 4). The four paths that showed decrease in connectivity strength with age were all matched with another path inputting into the same region that increased with age. These changes are progressive and, at least in some paths, show early onset. Specifically, four paths exhibited near monotonic increases or decreases (Table 6) from the initial (2nd) decade, with six of the eight paths demonstrating significant changes by the 3rd or 4th decade. This suggests that the process observed here is an intrinsic component of healthy, normal aging. The increases observed in the functional connectivity between regions also appeared to be unique to the DMN. In the control set of motor network regions, no connection between regions was observed to increase with age. This matches previous findings of decreases in the functional connectivity of the motor network in the resting state with age (Wu et al., 2007), and it was suggested that this may be a contributing factor to deteriorating motor ability with age. The changes in connectivity strength with age provide insight into future application of SEM modeling of disease effects for diagnostic purposes within individual subjects. Different cognitive disorders have implicated the DMN as a key network, and have highly differing average age of subjects (autism vs. Alzheimer's disease, for example). When considering deviation of connectivity strengths from expected norms, it is important to adjust for the effect of subject age on the expected connectivity strength of a path for a subject.

Changes in resting-state connectivity (including the DMN) with age have been previously demonstrated. Andrews-Hanna et al. (2007) found major decreases in DMN connectivity seeding from the MPFC as well as the precuneus, demonstrating some overlap with the findings of this study, including decreased connectivity between the precuneus and bilateral MTG. However, while within-network connectivity decreases with age, between-network connectivity has been shown to increase with age (Chan et al., 2014). This decrease in segregation and specialization may in part account for the paired increases and decreases in functional connectivity observed in this study. When parcellated by function, networks implicated in higher cognition demonstrated the most decrease in connectivity with age, while basic information processing networks showed an increase in connectivity with age (Geerligs et al., 2014). These studies also demonstrate changes in healthy aging, though applying their findings to this study remains a challenge as they examine the overall changes in multiple networks, rather than specific connectivity changes within a singular network.

Gray matter volume has been demonstrated to linearly decline across adulthood (Giorgio et al., 2010), and is a strong candidate for the source of reorganization of the DMN. Other structural changes that likewise exhibit a linear trend include declines in fractional anisotropy (from increase in perpendicular diffusivity but no change in parallel diffusivity) (Kochunov et al., 2007; Giorgio et al., 2010) as well as linear increases in mean diffusivity across adulthood (Giorgio et al., 2010). White matter volume (Giorgio et al., 2010) and ratio (Wu et al., 2013), however, was shown to have a mix of regions that linearly decrease with age or have a wide parabolic shape. While all tracts exhibit similar patterns or decline, they are not consistently the same across different tracts, with some demonstrating more change with age than others (Westlye et al., 2010). This may explain why not all paths demonstrate a change with age.

Common systemic disorders may also play a role in the progressive alterations of network activity observed here, particularly in late age. The present cohort did not exclude subjects with diabetes, hypertension or hypercholesterolemia, although other neurological and psychiatric disorders were excluded. Previous studies have suggested that long-standing hypertension reduces functional connectivity in regions supplied by the internal carotid artery (Mentis et al., 1994). Diabetes likewise can cause changes in functional connectivity in advance of frank microvascular damage (Duinkerken et al., 2009). Diabetes and hypercholesterolemia have also been shown to increase blood-brain barrier permeability that leads to amyloid deposition (Acharya et al., 2013), a pathology that has high specificity for the DMN (Vlassenko et al., 2010). All three of these common system disorders are associated with the gradual accumulation of white-matters lesions that could alter connectivity in the DMN, including compensatory increases (Sharp et al., 2011). The effect of these systemic disorders on DMN connectivity could not be assessed fully in the present study due to the limited sample size, but could be examined in future analysis with an expanded subject pool.

### Age invariant markers

Age-invariant paths also provide insight when considered in the context of previously observed disease effects. The strongest age-invariant path is between the PCC and pC. Yet, significant decrease in coherence (similarity of time series) between the posterior cingulate cortex and pC were observed in early Alzheimer's disease (He et al., 2007). Furthermore, both regions were frequently observed to show decreased metabolic activity in Alzheimer's disease (Minoshima et al., 1997; Johnson et al., 1998; Volkow et al., 2002), and easily could affect connectivity between the regions. This may indicate a useful marker that could predict early course of disease when significant declines are observed, and also suggests that the elderly subjects used in the present study are less likely to be suffering from not yet symptomatic mild cognitive impairment that has affected connectivity. Use of a per subject imaging measure as a marker for disease was previously demonstrated with decreased activity in the PCC and hippocampus that when fit to each subject was shown to be a potential marker to distinguish Alzheimer's disease from normal aging with 85% sensitivity and 77% specificity (Greicius et al., 2004). Abnormal changes in connectivity in the age-invariant paths could also likewise provide markers to be investigated.

### Plausibility of models

Overall, the exploratory SEM model showed very reasonable fits across the decade cohorts, and fit the subject pool very well as a whole. The Laird MACM model, while inferior in fit to the data across all fit statistics, still provided statistically significant paths that overlapped with the exploratory model. SEM models are often specified *a priori*, with a mask of ROIs that are specified to include the regions considered most interesting. By contrast, the ROIs were selected in this study using meta-analytic techniques pooling published literature. The *a priori* model used was also specified by meta-analytic connectivity modeling, and subsequent exploratory modeling only sought to optimize model fit. This creates a highly standardized method for defining and creating models, but it is worth considering whether the nodes and edges produced are plausible in the context of previous studies.

#### Node plausibility

The regions of interest selected were the strongest ALE determined regions from published deactivations. None of the included regions would be considered contentious, as all have been repeatedly demonstrated to be components of the DMN. (See Laird et al., 2009 for region-by-region discussion). The most notable deviation from common descriptions of the DMN is the absence of the hippocampus, which is often included in the definition of the DMN. Within the DMN, the hippocampus is functionally related to the posterior cingulate (Greicius et al., 2004; Teipel et al., 2010), and demonstrates structural atrophy significantly correlated with age (De Leon et al., 1997; Chowdhury et al., 2011). However, even at a more relaxed ALE criterion (expanding the number of nodes to 12), the hippocampus still did not achieve significance in ALE activation. As such, it was most appropriate to stay with the well-defined methodology for region selection and exclude the hippocampus from the analysis. However, given the significant age-related connectivity changes observed within the DMN, it may be worth exploring regions previously shown to be connected to the DMN and demonstrate significant age-related structural or functional changes in future analyses.

#### Edge plausibility (model overlap)

The Laird MACM model and the exploratory SEM model showed very good concordance, overlapping in 10 of 13 connections between regions (Figure 2). The extent of the overlap between models generated from meta-analytic techniques vs. primary resting state data is unsurprising, given previous studies have shown highly similar connectivity patterns are observed for co-activation (meta-analytic) and for resting-state connectivity (primary data) (Toro et al., 2008; Smith et al., 2009; Crossley et al., 2013). This phenomenon is observed throughout the brain, including but not limited to the DMN. The overlapping edges also have strong support in the structural imaging literature. Diffusion tensor imaging tractography has shown the PCC to be structurally connected the MPFG as well as to both MTG (Greicius et al., 2009). The same tractography study also found no evidence of a direct structural link between the MPFG to the bilateral MTG, reflecting similar findings to this study. Voxel-based connectivity show the pC/PCC, inferior parietal lobe, prefrontal gyrus, and left middle frontal gyrus show strong overlaps in both functional and structural connectivity to one another (Horn et al., 2014). Connectivity map of the ventral anterior cingulate showed the most significant functional connectivity with the MPFG and PCC (Greicius et al., 2003), in good agreement with both the Laird MACM model as well as the SEM exploratory model.

Models that are overly specified for a specific dataset are always a concern when performing fully exploratory SEM analyses. Considering the dramatic changes in connectivity in subjects from their 20 s to their 70 s, had we chosen to construct a fully exploratory model of either the youngest or the oldest cohort separately, they likely would have yielded somewhat different looking models for the same system. Given the strong overlap between the Laird MACM and exploratory SEM models, a meta-analytically constructed model may serve as a good starting model that can be subsequently refined to provide a model that is not as specifically tailored for the subject group being analyzed.

#### Model difference (hub shift)

However, while the Laird MACM model determined that the RMTG was the most extensively involved hub of the DMN, exploratory resting state modeling shows the precuneus to be the key hub of the network. Outside the differences between connections exiting the respective key hubs, the remaining connections in the Laird MACM model are identical to those in the exploratory model (though not necessarily in direction). Recent studies in voxel-based resting state connectivity have suggested that the precuneus is strong core hub of the DMN (Tomasi and Volkow, 2010, 2011; Utevsky et al., 2014), in agreement with the results of the exploratory model.

One possible source of the difference between the two hubs is that source of data for the Laird MACM model was task-based data. While DMN regions were determined by ALE using deactivations with task, coactivation patterns were determined based on task-driven co-increases. The underlying network structure does not appear to fundamentally change with cognitive state, only undergoing minor changes in line with previous studies showing that switching between rest and task will only switch a few pathways as required by cognitive demands (Goparaju et al., 2014). This reconfiguration of network coactivation patterns when shifting between rest and active task has also been observed in the parcellation of the cingulate cortex, another node of the DMN (Torta et al., 2013). Despite this switch in hub, however, the general structure of the network does not change and connectivity between the other regions remains undisturbed. This may explain why the strongest connection in the exploratory model, pC → PCC, was absent in the Laird MACM model. The posterior cingulate was determined to be the second strongest hub by both the Laird MACM model as well as the exploratory model. It may be that in the resting state, the precuneus is a key hub, and maintains strong communication with the posterior cingulate, a secondary hub. When transitioning to task, the precuneus may switch off as a hub while the right middle temporal gyrus turns on, and the communication between the precuneus and the posterior cingulate disappears. This possibility is supported by previous observation that the right middle temporal gyrus showed strong differentiation in functional connectivity when processing stories compared to rest, but not when listening to unrelated sentences compared to rest (Hasson et al., 2009). This effect was also observed more directly by Buckner et al. (2009), who noted that cortical hub locations were very similar when comparing active tasks vs. passive fixation, though the connectedness of prefrontal and temporal areas increase during task. Higher level cognitive processing is a part of many of the studies in the BrainMap database, and may explain the much more extensive right middle temporal gyrus connectivity found in the Laird MACM model. Despite the differences, the MACM model nevertheless demonstrated very similar patterns to connectivity found in primary resting state data, suggesting its application in guiding future modeling of functional networks. This is especially surprising given the widely discrepant source of data (published co-activations across tasks compared to primary resting state data), and may suggest that studies involving more similar tasks (such as a MACM model of finger tapping vs. primary finger tapping data) may yield even more similar models.

### Limitations and caveats

One limitation of the study is that the subjects were drawn from a randomly ascertained sample of ~ 30 extended Mexican-American families. Because of this, the subjects are much more genetically similar than a typical sample of healthy control subjects. That is, the findings could theoretically be specific only to regionally specific genetic and environment factors. To partially address this limitation, a second, non-overlapping sample of 105 subjects were selected for maximal genetic distance and analyzed in the same manner as reported here. This sample was more limited in age range, with the oldest subject being 54 years of age. Despite this, the cohort showed similar aging effects. To fully address this limitation, replication studies from other geographic regions with different genetic compositions and environmental factors will be needed.

Extending upon this, a second limitation of the study is that any exploratory model that any exploratory model constructed is specified by the attributes of the dataset including factors such as scan parameters and subject pool. While it is not expected that general connectivity patterns would differ wholesale across studies, specific values for connectivity strength may differ. This may create difficulties in projecting expected connectivity strengths across studies, and would merit investigation.

A third limitation of the study is the cross-sectional nature of the data. The model attempts to predict the course of connectivity changes with age without longitudinal data. However, given the range of ages the study attempts to model, longitudinal data of this magnitude would not be possible. Comparisons between cross-sectional and longitudinal designs have been performed previously, and have demonstrated that longitudinal studies may produce greater slopes of change (Desrosiers et al., 1998; Royall et al., 2005). Suggestions for the cause of this effect include cohort bias associated with cross-sectional design, and its influence on the results of this study may require further investigation.

### Conclusions and future directions

This study demonstrates progressive, bidirectional functional connectivity changes within the DMN in healthy aging, using SEM to quantify per-decade path coefficients. Declining path coefficients and rising path coefficients were observed to share common nodes, suggesting a compensatory mechanism for maintaining node input. This provides a framework for interpreting changes in DMN integrity in brain disorders across the age range and for assessing the effects of systemic disorders implicated in accelerated brain aging. Per-subject biometrics have the potential to be used as biomarkers for genetic analyses, for diagnosis, for prediction of response to therapy, and the like. For example, if this analysis was applied to the entire GOBS cohort, path coefficients could be used a biomarkers for gene discovery. Similarly, the extensive neuropsychological data available on this cohort, correlations between individual path coefficients and psychometric scores could provide guide functional interpretation of the individual paths.

Furthermore, the study demonstrates high correspondence between a meta-analytically derived model and a fully optimized exploratory model, suggesting meta-analytic modeling as a generally applicable method for constraining analysis of brain networks. Meta-analytic data may be a good source to generate starting models that can be refined and used to investigate network connectivity and disease effects in other systems as well.

## Author contributions

KL is the primary author. AL made major edits and proposed the project. LP was responsible for designing the analysis structure as well writing part of the statistical section. DM, JB, and DG each made edits and revisions to polish the manuscript. PF is the corresponding author who oversaw the project and made the most edits and revisions to the manuscript.

### Conflict of interest statement

The authors declare that the research was conducted in the absence of any commercial or financial relationships that could be construed as a potential conflict of interest.

## Abbreviations

ALE: Activation Likelihood Estimation
DCM: Dynamic Causal Modeling
DMN: Default Mode Network
MACM: Meta-Analytic Connectivity Modeling
SEM: Structural Equation Modeling.
