# Supplementary material for: Progressive Bidirectional Age-Related Changes in Default Mode Network Effective Connectivity across Six Decades
Source: Front Aging Neurosci. 2016 Jun 14;8:137. doi: 10.3389/fnagi.2016.00137 (PMC4905965; doi:10.3389/fnagi.2016.00137)
Supplement: Supplementary file 1 [file Table1.docx]

| **Decade** | **Absolute Displacement (mm)** | **Relative Displacement (mm)** |
| --- | --- | --- |
| 20s | 0.106 + 0.009 | 0.067 + 0.008 |
| 30s | 0.104 + 0.012 | 0.071 + 0.010 |
| 40s | 0.111 + 0.008 | 0.079 + 0.009 |
| 50s | 0.098 + 0.008 | 0.066 + 0.005 |
| 60s | 0.111 + 0.013 | 0.083 + 0.014 |
| 70s | 0.119 + 0.014 | 0.087 + 0.011 |

Supplementary Table I: Absolute and relative motion displacement between TRs for each decade. Absolute displacement is average head displacement compared to a reference time point (middle time point) while relative displacement is average head displacement between sequential time points. There are no significant differences across groups based on ANOVA nor is there a significant trend based on Pearson r.
