# Supplementary material for: Progressive Bidirectional Age-Related Changes in Default Mode Network Effective Connectivity across Six Decades
Source: Front Aging Neurosci. 2016 Jun 14;8:137. doi: 10.3389/fnagi.2016.00137 (PMC4905965; doi:10.3389/fnagi.2016.00137)
Supplement: Supplementary file 2 [file Table2.docx]

| **Path** | **Path Coefficient (By Decade)** | **Pearson r Correlation with Decade** |
| --- | --- | --- |
| **pC 🡪 LMFG** | **0.22 \| 0.16 \| 0.10 \| 0.12 \| 0.09 \| 0.08** | **-0.90** |
| **pC 🡪 RIPL** | **0.08 \| 0.07 \| 0.05 \| 0.13 \| 0.14 \| 0.15** | **0.82** |
| pC 🡪 LIPL | 0.14 \| 0.13 \| 0.08 \| 0.09 \| 0.18 \| 0.11 | 0.01 |
| **pC 🡪 LMTG** | **0.31 \| 0.20 \| 0.17 \| 0.16 \| 0.13 \| 0.06** | **-0.95** |
| pC 🡪 PCC | 0.44 \| 0.42 \| 0.39 \| 0.35 \| 0.44 \| 0.43 | -0.05 |
| **PCC 🡪 LMTG** | **0.23 \| 0.28 \| 0.33 \| 0.30 \| 0.33 \| 0.35** | **0.88** |
| **PCC 🡪 RMTG** | **0.14 \| 0.09 \| 0.23 \| 0.21 \| 0.24 \| 0.24** | **0.80** |
| PCC 🡪 MPFG | 0.25 \| 0.34 \| 0.34 \| 0.23 \| 0.25 \| 0.24 | -0.45 |
| PCC 🡪 vACC | 0.07 \| 0.14 \| 0.18 \| 0.12 \| 0.07 \| 0.05 | -0.39 |
| **MPFG 🡪 vACC** | **0.37 \| 0.30 \| 0.26 \| 0.19 \| 0.13 \| 0.20** | **-0.88** |
| **MPFG 🡪 LMFG** | **0.20 \| 0.24 \| 0.26 \| 0.28 \| 0.31 \| 0.30** | **0.95** |
| **LIPL 🡪 RIPL** | **0.39 \| 0.28 \| 0.16 \| 0.22 \| 0.12 \| -0.01** | **-0.94** |
| **LMTG 🡪 RMTG** | **0.40 \| 0.31 \| 0.25 \| 0.23 \| 0.19 \| 0.19** | **-0.94** |

Supplementary Table II: Path coefficients (per decade cohort) for the exploratory SEM model using data that has regressed out confounds using white matter and CSF signal. Bolded paths demonstrate significant linear trends with age. Compared with the data that did not regress out physiological noise using white matter and CSF signals, the trends largely hold the same. The only difference is that MPFG 🡪 vACC is now a path with significant linear decline with age. Standard errors range from 0.014 to 0.021.
